# Population-specific diversity of the immunoglobulin constant heavy G chain (IGHG) genes

**DOI:** 10.1038/s41435-021-00156-2

**Published:** 2021-12-04

**Authors:** Arman A. Bashirova, Wanjing Zheng, Marjan Akdag, Danillo G. Augusto, Nicolas Vince, Krista L. Dong, Colm O’hUigin, Mary Carrington

**Affiliations:** 1 Basic Science Program, Frederick National Laboratory for Cancer Research in the Laboratory of Integrative Cancer Immunology, National Cancer Institute, Bethesda, MD USA; 2 The Laboratory of Integrative Cancer Immunology, National Cancer Institute, Bethesda, MD USA; 3 Programa de Pós-Graduação em Genética, Universidade Federal do Paraná, Curitiba, Brazil; 4 Department of Neurology, University of California San Francisco, San Francisco, CA USA; 5 Université de Nantes, CHU Nantes, Inserm, Centre de Recherche en Transplantation et Immunologie, UMR 1064, ITUN, F-44000 Nantes, France; 6 Females Rising through Education, Support, and Health, Durban, KwaZulu-Natal South Africa; 7 Ragon Institute of MGH, MIT and Harvard, Cambridge, MA USA

**Keywords:** Structural variation, Immunogenetics

## Abstract

Human immunoglobulin G (IgG) molecules, IgG1, IgG2 and IgG3, exhibit substantial inter-individual variation in their constant heavy chain regions, as discovered by serological methods. This polymorphism is encoded by the *IGHG1*, *IGHG2*, and *IGHG3* genes and may influence antibody function. We sequenced the coding fragments of these genes in 95 European Americans, 94 African Americans, and 94 Black South Africans. Striking differences were observed between the population groups, including extremely low amino acid sequence variation in IGHG1 among South Africans, and higher IGHG2 and IGHG3 diversity in individuals of African descent compared to individuals of European descent. Molecular definition of the loci illustrates a greater level of allelic polymorphism than previously described, including the presence of common IGHG2 and IGHG3 variants that were indistinguishable serologically. Comparison of our data with the 1000 Genome Project sequences indicates overall agreement between the datasets, although some inaccuracies in the 1000 Genomes Project are likely. These data represent the most comprehensive analysis of IGHG polymorphisms across major populations, which can now be applied to deciphering their functional impact.

## Introduction

**Fig. 1:**
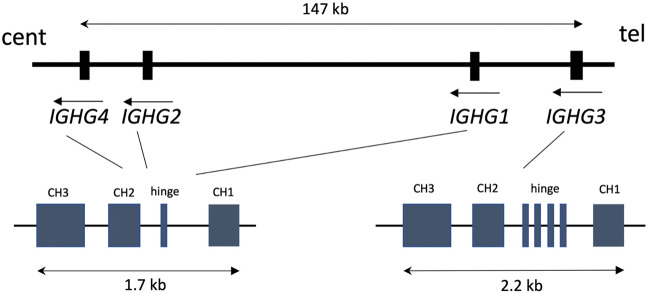
Schematic map of the *IGHG* locus. The *IGHG* genes have similar exon/intron structure, except for *IGHG3*, which has multiple hinge exons. The map is drawn based on the human genome assembly (GRCh38.p13) and transcript data extracted using the Ensembl browser (www.ensembl.org).

Human IgG subclasses, IgG1, IgG2, IgG3, and IgG4, display distinct functional properties due to the differences in their constant heavy chains, which consist of CH1, CH2, and CH3 domains and a hinge region between the CH1 and CH2 domains [1]. These fragments contain binding sites for complement component C1q and Fc gamma receptors [2, 3], which regulate antibody effector functions, and the neonatal Fc receptor (FcRn), controlling antibody transport and half-life [4]. The constant heavy chains of the four IgG subclasses are encoded by distinct genetic loci, *IGHG1*, *IGHG2*, *IGHG3*, and *IGHG4* (Fig. 1) [5]. These genes form a cluster spanning a ~150 kb region within the Ig heavy chain constant (*IGHC*) locus on human chromosome 14. The CH1, hinge, CH2 and CH3 domains of each gene are encoded by separate exons. However, *IGHG3* is unique in having varying number of hinge exons, ranging from two to four across individuals [6].

Sequence differences in constant heavy chains exist not only between the IgG subclasses, but also within the subclasses, originally discovered using serological methods [7]. Based on antigenic properties, IgG allotypes were classified using the Gm (Gamma marker) nomenclature, where G1m, G2m, and G3m allotypes designate IgG1, IgG2, and IgG3, respectively. The Gm allotypes across these three subclasses were observed to be inherited in certain frequent combinations, reflecting a high level of linkage disequilibrium (LD) between the corresponding loci. Genetic polymorphism underlying the serological Gm diversity was further characterized within each of the *IGHG1*, *IGHG2*, and *IGHG3* genes [1, 7]. Multiple alleles of these genes are annotated in the IMGT database, including 14 *IGHG1*, 17 *IGHG2*, and 29 *IGHG3* alleles encoding 6, 7, and 21 distinct protein variants, respectively, as of August 2021 (www.imgt.org). Differences between alleles include single nucleotide polymorphisms (SNPs) as well as variation in the number of *IGHG3* hinge exons. These data show that *IGHG* variation involves more amino acid positions than those identified serologically.

Functional consequences of *IGHG* polymorphism are largely unknown, but a few have been elucidated. An IGHG3 variant with histidine at position 435 (435H) in the CH3 domain was shown to have prolonged half-life compared to that containing the arginine variant (435 R) due to higher affinity of the IgG3-435H variant to FcRn at low pH [4]. Differential FcRn binding has also been detected for IgG1 allotypes, which are fixed for the 435H variant, but differ from one another at positions 214 (CH1) and/or 356/358 (CH3) [8]. Polymorphisms at residues 291, 292 and 296 in the IgG3 CH2 domains have recently been shown to influence antibody dependent cellular cytotoxicity (ADCC) in a comprehensive study that examined 27 genetically defined IgG allotypes [9]. In addition, antibody effector functions can be modulated by length of the IgG3 hinge [9, 10]. Besides the direct effect of allotypic variation on the antibody structure and function, certain variants in the CH domains may be in LD with polymorphisms that regulate IgG subclass switching, possibly explaining the variation in IgG subclass serum levels observed in carriers with certain IgG allotypes [1, 11–13].

The biological importance of *IGHG* polymorphism is suggested by multiple disease associations with this locus [14–16]. From a clinical standpoint, the potential immunogenicity of therapeutic antibodies when their allotypes differ from the patient’s endogenous allotypes may result in poor response to these therapies, although evidence is lacking in this regard [1].

IgG allotypes have been studied extensively using serological methods and were found to display population-specific frequency distributions [7, 17, 18]. However, the corresponding genetic information is limited. A recent study by Calonga-Solis et al. [19] described polymorphism in the *IGHG* genes in Brazilian populations, including Amerindians, Japanese- and Euro-descendants, but notably, people of African descent were not considered. Here, we characterized *IGHG1*, *IGHG2*, and *IGHG3* coding regions polymorphism in three population groups, including African Americans (AA, *N* = 94), European Americans (EA, *N* = 95) and Black South Africans (SA, *N* = 94). As a validation, we compared our genotyping data with that available from the 1000 Genomes Project (1kGP). These data lay a foundation for characterizing the functional consequences for IGHG variation and its potential impact on disease and therapy outcomes.

## Results

### IGHG SNPs and haplotypes

We amplified *IGHG1, IGHG2*, and *IGHG3* genomic fragments, including exons encoding the CH1, hinge, CH2 and CH3 domains (Fig. 1), and sequenced the PCR products using Sanger methodology in order to identify polymorphic sites. The fragment encompassing the *IGHG3* hinge exons was amplified using primers outside of the ~200 bp repetitive element (Fig. S1) and the number of exons in each person was determined based on electrophoretically-defined PCR fragment length. SNPs in the *IGHG3* hinge exons were not considered because of the inability to read sequences directly upon heterozygosity for exon copy number. Our analysis also did not include the *IGHG4* gene given the previously reported copy number variation at this locus [20].

Sequence analysis of the coding regions in the three genes revealed a total of 87 SNPs across the three populations sampled in our study (Table S1). These data were compared to publicly available whole genome sequences by extracting variants in corresponding regions from two 1kGP datasets: phase 3 [21] (1kGP-ph3) and the newly assembled high coverage version, 1kGP-30X [22]. Of 87 SNPs detected in our cohorts, 71 were present in 1kGP-ph3, and 83 were detected in 1kGP-30X (Table S1). While the vast majority of the SNPs that were present in one dataset and not the other were of low frequency, there were several common SNPs that were discrepant in this regard (Table S1). For example, variants at positions 161 and 311 in CH3 exons of *IGHG2* and *IGHG1*, respectively, were present in our dataset and 1kGP-30X (16–29% allelic frequency), but absent in 1kGP-ph3. On the other hand, 1kGP-30X detected SNPs at positions 168 and 313 of *IGHG1* CH3 and *IGHG2* CH3, respectively (19–24% allelic frequency), and these SNPs were not found in our dataset nor 1kGP-ph3. The possibility of errors in the 1kGP datasets with regard to common SNPs is evident when comparing 1kGP data to that in the IMGT for *IGHG* alleles, which represent cDNA clones deposited by various research groups (Fig. S2). The common SNPs missed in 1kGP-ph3 and detected in our cohorts are present among the IMGT alleles. Further, SNPs present in 1kGP-30X with frequency >3%, but missing in our data are also missing in IMGT. Thus, our genotypes more closely reflect those compiled in the IMGT dataset than does either version of the 1kGP datasets.

We used Haploview software to phase the identified SNPs and predict *IGHG* alleles, which we then matched to alleles annotated in the IMGT database (Tables S2–S4). Several putatively new alleles were identified, with allelic frequencies of up to 12%. Alleles that were very common in one population were often much rarer in another; for example, IGHG1*03, IGHG2*03, and IGHG3*11 were common in EA, but less so in AA and SA, and IGHG1*02, IGHG2*06, and IGHG3*01 were common in AA and SA, but less so (or missing altogether) in EA. Thus, *IGHG* allelic frequencies demonstrated remarkable population-specific distributions.

### IGHG allotypes

IGHG allotypes (i.e., protein variants) were estimated by phasing only amino acid changing SNPs in our dataset. In order to compare our data to the 1kGP data, we extracted phased non-synonymous *IGHG* SNP data from 1kGP-ph3 for three super-populations, African (AFR, *N* = 661), European (EUR, *N* = 503), and East Asian (EAS, *N* = 504), and considered variants present with frequencies >1%. As indicated above, a number of discrepancies were evident between both versions of the 1kGP data and our data. We focused here only on the older 1kGP-ph3 because the 1kGP-30X data contains several SNPs with high frequencies that are likely to have been assigned to *IGHG* genes inaccurately, and it is preferable to analyze a dataset that may miss some SNPs rather than one that contains falsely assigned variants. Alleles encoding the same amino acid sequence (i.e., alleles that differed only by synonymous changes) were grouped together and given an allotype name that corresponds to the most frequent allele in that grouping. For instance, the allotype IGHG1*02 is encoded by multiple *IGHG1* alleles that differ only by synonymous changes (IGHG1*02, *05, *09, etc.; Table S2).

The most frequent IGHG1 allotypes differed across population groups: IGHG1*03, IGHG1*02, and IGHG1*08, in individuals of European descent (EA and EUR; IED), individuals of African descent (AA, SA and AFR; IAD), and EAS, respectively (Table 1). Strikingly, IGHG1*02 was nearly fixed in the SA sample and it was also the most common IGHG1 variant across populations overall. The most frequent EAS allotype, IGHG1*08, could be a product of allelic gene conversion between IGHG1*02 and IGHG1*03 (Table 1).

**Table 1:** Estimated frequencies of IGHG1 allotypes^1^.

		CH1			CH2		CH3				Freq. this study			Freq. 1kGP-ph3			Global
IGHG1	Gm	131	199	214	280	291	356	358	422	431	EA	AA	SA	AFR	EUR	EAS	freq.
*02	17, 1	S	I	K	D	P	D	L	V	A	0.163	0.760	0.989	0.943	0.204	0.315	0.562
*03	3	-	-	R	-	-	E	M	-	-	0.700	0.165		0.015	0.516	0.013	0.235
*04	17, 1, 27	-	-	-	-	-	-	-	I	-		0.016					0.003
*07	17, 1, 2	-	-	-	-	-	-	-	-	G	0.126	0.021		0.004	0.068	0.095	0.052
*08	3, 1	-	-	R	-	-	-	-	-	-		0.011		0.011	0.083	0.520	0.104
*n3	17, 21, 1, 27	-	-	-	-	L	-	-	I	-		0.011					0.002
*n4	17, 1	-	-	-	E	-	-	-	-	-				0.011			0.002
*1K1	17	-	-	-	-	-	E	M	-	-				0.009	0.042	0.007	0.010
*1K2	17, 2	-	-	-	-	-	E	M	-	G					0.036		0.006
*1K3	3	C	-	R	-	-	E	M	-	-				0.002	0.019		0.004
*1K4		-	T	R	-	-	E	M	-	-					0.026		0.004
*1K5		-	T	R	-	-	-	-	-	-					0.001	0.024	0.004
IGHG2		C	T	T	D	P	E	M	V/I	A							
IGHG3		C	T	R	D	P/L	E	M	I/V	A							
IGHG4		C	T	R	D	P	E	M	V	A							

^1^Numerical allotype names correspond to IMGT nomenclature (Tables S2–S4), letter “n” designates newly identified allotypes in this study, and allotypes found only in 1kGP-ph3 dataset are labeled “1K”. Amino acid positions are shown in accordance with the Eu numbering system. Allotypes are listed in bold when present at frequencies >10% in at least one group, and amino acid positions are bolded when distinguished by serological typing. Gm allotypes were defined according to Lefranc and Lefranc [7]. EA – European Americans (*N* = 95), AA – African Americans (*N* = 94), AFR – Africans (*N* = 661), EUR – Europeans (*N* = 503), EAS – East Asians (*N* = 504). Allotypes with estimated frequencies >1% are shown.

IGHG2 allotypes demonstrated higher level of diversity than IGHG1 with IGHG2*02, IGHG2*03 and IGHG2*06 observed most frequently (Table 2). While IGHG2*03 was common in all six populations, IGHG2*02 was relatively rare among IAD and IGHG3*06 was absent among IED. A new allotype, IGHG2*n3, was identified in the AA and SA populations at fairly high frequencies, but was strikingly absent in the AFR sample of 1kGP; this allotype differs from IGHG2*03 by a single amino acid at position 422 in the CH3 domain (V/I, Table 2), and is likely to be a product of gene conversion between *IGHG2* and *IGHG3* (Tables 2 and 3). The absence of IGHG2*n3 in the 1kGP-ph3 could be a result of incorrect sequence assembly, which did not identify the 422V/I polymorphism at this locus (G2_CH3_243, Table S1).

**Table 2:** Estimated frequencies of IGHG2 allotypes^1^.

		CH1				CH2		CH3				Freq. this study			Freq. 1kGP-ph3			Global
IGHG2	G2m	189	192	193	213	282	309	378	392	412	422	EA	AA	SA	AFR	EUR	EAS	freq.
*02	23	T	N	F	K	M	V	A	K	V	V	0.447	0.112		0.015	0.332	0.510	0.236
*03	..	P	-	-	-	V	-	-	-	-	-	0.532	0.457	0.341	0.639	0.451	0.440	0.477
*06	..	P	-	-	-	V	-	S	-	-	-		0.245	0.385	0.304			0.156
*11	..	P	-	-	-	V	L	-	-	-	-			0.029	0.013		0.003	0.008
*15	..	P	-	-	-	V	-	-	N	-	-		0.011		0.012	0.001	0.002	0.004
*n1	23	-	S	L	-	-	-	-	-	-	-	0.011						0.002
*n3	..27	P	-	-	-	V	-	-	-	-	I		0.170	0.133				0.051
*n9	..	P	-	-	-	V	M	-	-	-	-			0.045				0.008
*n10	..	P	-	-	E	V	M	-	-	-	-			0.029				0.005
*n12	..	P	-	-	E	V	M	-	-	M	-			0.016				0.003
*1K1	23	P	-	-	-	-	-	-	-	-	-				0.002	0.104	0.024	0.022
*1K2	..	-	-	-	-	V	-	-	-	-	-				0.002	0.102	0.021	0.021
IGHG1		P	S	L	K	V	L	A	K	V	V/I							
IGHG3		P	S/N	L/F	K	V	L/V	A	K/N	V	I/V							
IGHG4		P	S	L	K	V	L/V	A	K	V	V							

^1^See footnote to Table 1.

**Table 3:** Estimated frequencies of IGHG3 allotypes^1^.

		CH1			CH2							CH3									Freq. this study			Freq. 1kGP-ph3			Global
IGHG3	G3m	192	193	H^2^	234	274	291	292	296	327	339	379	384	392	397	409	419	422	435	436	EA	AA	SA	AFR	EUR	EAS	freq.
*01	5*	S	L	4	L	Q	P	R	Y	A	T	V	S	N	M	K	Q	I	R	F		0.392	0.459	0.328	0.007	0.364	0.258
*03	24*	-	-	3	-	-	-	-	-	-	-	-	-	-	V	R	E	V	-	-		0.169	0.140				0.052
*07	5*	-	-	-	-	-	-	-	-	-	-	-	-	K	-	-	-	-	-	-		0.090	0.050	0.073			0.036
*08	5, 14, 26, 27	-	-	-	-	-	-	-	-	-	-	-	N	-	-	-	-	-	-	-				0.009		0.023	0.005
*11	5*	-	-	-	-	-	-	-	F	-	-	-	-	-	-	-	-	-	-	-	0.626	0.147		0.015	0.411	0.009	0.201
*12	5*	-	-	3	-	-	-	-	F	-	-	-	-	-	-	-	-	-	-	-	0.037	0.011					0.008
*13	6*	-	-	-	-	-	-	-	-	-	-	-	-	K	-	-	E	-	-	-		0.037	0.072				0.018
*14	21*	-	-	-	-	-	L	-	-	-	-	-	N	-	-	-	-	-	-	Y	0.253	0.053	0.045	0.012	0.178	0.223	0.127
*15	21*	-	-	-	-	-	L	-	-	-	-	-	N	K	-	-	-	-	-	Y				0.003	0.003	0.017	0.004
*16	21*	-	-	-	-	-	L	-	-	-	A	-	N	-	-	-	-	-	-	Y	0.032	0.016			0.033		0.014
*17	15*	N	F	3	-	-	-	-	-	-	-	M	-	K	V	-	-	-	H	Y		0.032	0.059	0.025			0.019
*19	16*	-	-	3	-	-	-	W	-	-	-	M	-	K	V	-	-	-	H	Y					0.002	0.056	0.010
*22	21, 27	-	-	-	-	-	L	-	-	-	-	-	N	-	-	-	-	-	H	Y					0.017	0.030	0.008
*25	21*	-	-	-	-	K	L	-	-	-	-	-	N	-	-	-	-	-	-	Y						0.012	0.002
*26	5*	-	-	-	-	-	-	-	F	G	-	-	-	-	-	-	-	-	-	-	0.037						0.006
*n3	5*	-	-	-	F	-	-	-	-	-	-	-	-	-	-	-	-	-	-	-			0.063				0.011
*01m	6*	-	-	-	-	-	-	-	-	-	-	-	-	-	-	-	E	-	-	-			0.044				0.007
*n5	15*	-	-	3	-	-	-	-	-	-	-	M	**-**	K	V	-	-	-	H	Y			0.037				0.006
*n7	5, 14, 21, 26, 27	-	-	-	-	-	L	-	-	-	-	-	N	-	-	R	-	-	-	-			0.016				0.003
*1K1	5, 11, 13, 24, 26	-	-		-	-	-	-	-	-	-	-	-	-	V	R	-	V	-	-				0.103			0.017
IGHG1		S	L		L	K	P/L	R	Y	A	A	V	N	K	V	K	Q	V/I	H	Y							
IGHG2		N/S	F/L		P	Q	P	R	F	G	T	V	N	K/N	M	K	Q	V/I	H	Y							
IGHG4		S	L		F	Q	P	R	F	G	A	V	N	K	V	R/K	E	V	H	Y							

^1^See footnote to Table 1. Table 3 does not include IGHG3*1K2-1K17, which are shown in Table S5. IGHG3*01m allotype was described by Richardson et al. [27].

^2^H – number of hinge exons. The number of exons encoding the hinge was determined in EA, AA, and SA, but for the 1kGP haplotypes it was assumed based on matching IMGT allotypes. Whereas all unique amino acid sequences in CH1/CH2/CH3 were associated with a specific hinge length (3 or 4 exons), the IGHG3*11 and IGHG3*12 only differed in the number of hinge exons. Therefore, some allotypes determined as IGHG3*11 in AFR, EUR, and EAS could be IGHG3*12.

A single trimorphism (V/L/M) was detected at position 309 of IGHG2 (G2_CH2_231), where the 309M variant was found exclusively in SA. Interestingly, one SA individual possessed three nucleotide variants at the corresponding position, suggesting a gene duplication of the *IGHG2* locus (Fig. S3). Although the gene duplication may have been present in more individuals, this is likely to be a rare event since the SNP data were, overall, consistent with Hardy-Weinberg equilibrium.

The IGHG3 allotypes demonstrated the highest level of diversity among the three loci (Table 3). IGHG3*11 is the most common allotype in IED, whereas IGHG3*01 is the most common allotype in IAD and EAS. These two allotypes differ only at position 296 (Y/F) and cannot be distinguished serologically. IGHG3*03 was not present in the 1kGP-ph3 dataset, which misses SNP encoding the IGHG3 419Q/E polymorphism, likely due to sequence assembly error (G3_CH3_234, Table S3).

The IGHG3 allotypes containing histidine at position 435, which is known to increase the IgG3 half-life and facilitate placental transport [4], were rare in our dataset (IGHG3*17 and IGHG3*n5). The corresponding SNP rs4042056 (G3_CH3_283) was not detected in our EA sample (i.e., fixed for arginine at this position), but was present at frequencies of 3% and 10% among the AA and SA individuals, respectively (Table S1). This SNP was detected at higher frequency in the 1kGP-ph3 dataset (10–16% in EUR, AFR and EAS) giving rise to multiple haplotypes (Table 3 and S5).

The presence of four *IGHG3* hinge exons was by far the most common length variant across the genotyped populations followed by presence of three exons, which showed higher allelic frequency in AA/SA compared to EA (Table 4). We have also detected single individuals carrying an allele with two exons and an allele with five exons in an AA and an EA individual, respectively. For the majority of allotypes identified in our study, the hinge length can be unambiguously assigned using the known IMGT alleles and the CH domain sequences. The exception is distinguishing IGHG3*11 from IGHG3*12, which are identical in the CH domains, but possess hinge domains encoded by four vs. three exons, respectively.

**Table 4:** *IGHG3* hinge exon copy number variation (allele frequency).

# hinge exons	Length, aa	EA (*N* = 95)	AA (*N* = 94)	SA (*N* = 94)
2	32		0.005	
3	47	0.037	0.234	0.239
4	62	0.958	0.761	0.761
5	77	0.005		

Overall, frequencies of the allotypes encoded by the three *IGHG* genes demonstrated a high level of diversity across populations, and support genetic admixture in the AA population. While providing new details, the data were consistent with previous serological and genotypic findings.

### LD between the IGHG genes

**Fig. 2:**
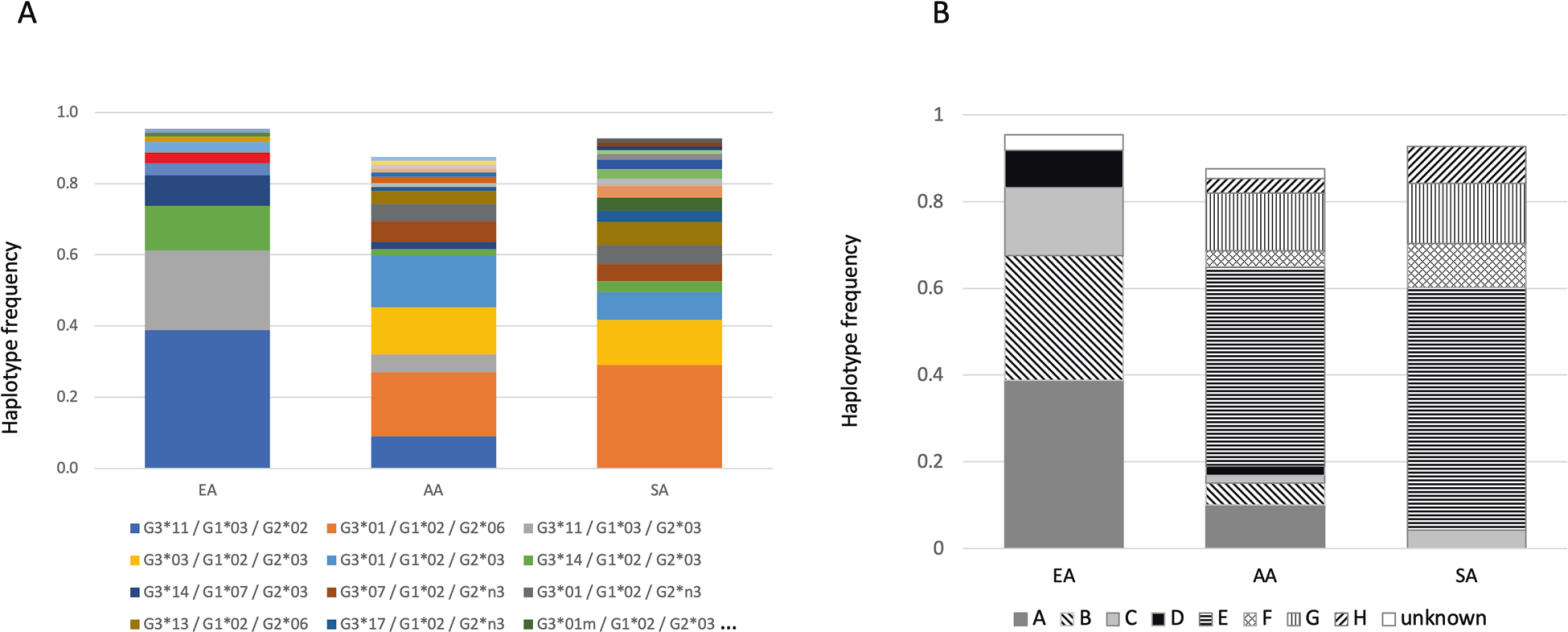
IGHG3_IGHG1_IGHG2 haplotypes estimated in three populations with frequency >1%. **A** Haplotypes estimated using genetic data of non-synonymous SNPs. Allotypes for each locus represent unique amino acid sequences as shown in Tables 1–3. **B** Gm haplotypes corresponding to the genetic haplotypes according to Lefranc and Lefranc [7]. The data for these graphs is shown in Tables S6 and S7.

Serological data implicate a high level of LD between the *IGHG* genes [7], and to test this in our dataset, multigenic haplotypes were generated using Haploview based on amino acid changing variants (Fig. 2, S4 and Tables S6–S7). There were 10 haplotypes in the EA group and 18 haplotypes in each of the AA and SA groups restricting to those observed at frequency of >1%. Haplotype frequencies differed substantially between population samples with no common haplotypes observed between EA and SA except for one (Fig. 2A, Table S6). This is a reflection of the differential allotype frequencies across the groups. Nine haplotypes were shared by the AA and SA groups, and four haplotypes were shared between AA and EA, highlighting admixture between these two groups. The haplotypes were further categorized using serological Gm nomenclature [7] (Fig. 2B, Table S7). The corresponding frequencies were concordant with previously published European and South African data [7, 17, 18].

### Inter-population differentiation

The apparent population-specific distribution of *IGHG* alleles prompted us to evaluate their differentiation by calculating fixation indices (F_ST_), which measures inter-population variance of allele frequencies over an average diversity of populations [23]. Large F_ST_ values indicate inter-population differentiation and suggest the possibility of natural selection. We calculated pairwise F_ST_ of *IGHG1*, *IGHG2*, and *IGHG3* non-synonymous SNPs with global minor allele frequencies >10% in the AFR, EUR and EAS super-populations from 1kGP-ph3 and compared them with the value distribution of a genome-wide sample of SNPs in the same populations (Fig. S5). Four SNPs demonstrated significantly high F_ST_ values in pairwise comparisons (Table 5). Whereas all four SNPs showed significantly high F_ST_ values in the AFR-EUR comparisons, none were significant in the AFR-EAS analysis; three of the four were significantly high in the EUR-EAS comparison. The F_ST_ pattern is consistent with the breakdown of the strong LD between the *IGHG1* SNPs encoding amino acid positions 214, 356 and 358 in the EAS super-population. Strong LD across these three amino acids in *IGHG1* is characteristic of IED and IAD, whereas breakdown between positions 214 and 356 forming the common IGHG1*08 allotype is characteristic of EAS (Table 1).

**Table 5:** Non-synonymous *IGHG* SNPs with significantly high F_ST_ values in pairwise analysis of the AFR, EUR and EAS populations from the 1kGP data^1^.

rs #	IGHG	AA pos. (Eu)	Assoc. alleles	F_ST_ AFR-EUR	F_ST_ AFR-EAS	F_ST_ EUR-EAS
rs1071803	IGHG1	214	*03/08	**0.628**	0.5514	0.01
rs1045853	IGHG1	356	*03	**0.623**	0	**0.606**
rs11621259	IGHG1	358	*03	**0.625**	0	**0.607**
rs12890621	IGHG3	296	*11/12	**0.619**	0.0011	**0.607**

^1^In bold – significant F_ST_ values.

## Discussion

IgG allotype diversity has been characterized across various populations by serological methods [7, 17, 18], whereas characterization of polymorphisms and their frequencies in the corresponding *IGHG* genes remains sparse due to the homology between the genes and related difficulties in analyzing genotypes. Here, we determined *IGHG* variation in three population groups from the U.S. and South Africa, representing the first comprehensive genetic analysis of the *IGHG* locus in IAD, and compared these to the 1kGP data. Overall, our data were in agreement with the 1kGP data, but certain distinctions were observed. These included a few common SNP discrepancies between the two datasets and greater diversity of *IGHG* alleles in the 1kGP dataset than in ours. There are likely several causes of these discrepancies. The 1kGP data is obtained using next generation sequencing (NGS), which relies on assembly of short overlapping reads (~100 bp) that can phase variants inaccurately [24], particularly in regions of high sequence similarity, such as the *IGHG* genes. These issues probably resulted in drop-out of low-confidence SNPs in 1kGP-ph3, and erroneous mapping in the less conservative approach of 1kGP-30X. Although our data may be prone to allele drop-out if a variant occurs within the site of primer annealing, it is strengthened by sequencing of long genomic fragments (600–1800 bp) derived from gene-specific amplification, which increases confidence in accuracy of gene-specific calling of the variants. Of note, with regard to common SNPs that were discrepant between our data and the 1kGP datasets, we found that our frequencies were generally closer to that of gnomAD data generated by NGS (http://www.ensembl.org). Further, population substructure differentiating EUR from EA, or AFR from AA and SA may impact genotypic differences across these populations. Additional limitations of our study that could have affected the data include relatively small sample sizes that would miss rare variants as well as potential population substructure within the groups that may influence haplotype estimation. However, the consistency of our data with Hardy-Weinberg equilibrium, and its general concurrence with 1kGP data, IMGT allelic variation, and published serological data point to accuracy in our genotyping data of the *IGHG* genes. Importantly, these consistencies endorse the authenticity of the extended information regarding genetic variation at the *IGHG* locus provided by our genotyping.

Some differences observed across populations involve variants that may influence antibody function. The IGHG3 435H variant, which associates with prolonged half-life of the antibody due to efficient FcRn binding [4], has been found at higher frequency in IAD and EAS compared to IED. The most frequent IGHG1 allotype IGHG1*08 in EAS corresponds to Gm3,1, which showed the strongest interaction with FcRn [8], whereas the most frequent IGHG1 allotypes in IAD and IED correspond to the intermediate and weaker binders, Gm17,1 (IGHG1*02) and Gm3 (IGHG1*03), respectively (Table 1). IGHG1*02 and IGHG1*03 also differentially bind to decoy FcRs from HSV-1 [25] and HCMV [26], which may potentially underlie disease associations [15, 16]. IGHG3 allotypes with a leucine at position 291 (291L), a tryptophan at position 292 (292W), and a phenylalanine at position 296 (296F) have been found to exhibit reduced ADCC [9]. The 291L and 296F allotypes were present at higher frequencies in IED than in IAD, and the 292W was detected only in 1kGP-ph3 data at low frequencies in EUR and EAS. Given the observed frequency patterns, IAD may possess more potent IgG3 allotypes than IED. Notably, among the functionally distinct allotypes are those defined by SNPs that demonstrated significant inter-population differentiation by F_ST_ analysis (Table 5), suggesting that these SNPs may be under selection pressure due to their functional consequences.

Another potentially functional polymorphism that is differentially present across populations, is the IGHG3 hinge length variation. The most common variant is encoded by four exons, and the second most common variant is encoded by three exons, which is present more frequently among IAD than IED (Table 4). Chu et al. [10] suggested that decreasing the hinge length diminishes phagocytic activity of IgG3 based on studies using hinge variants on the backbone of the IGHG3*01 allotype. However, our genotyping data indicate that the three-exons variant does not occur on a IGHG3*01 backbone, but rather, is present primarily in an IGHG3*03 allotype (Table 3). De Taye et al. [9] tested only natural variants for ADCC activity and did not detect clear difference between three- and four-exon hinge allotypes, but the IGHG3*04 allotype with shorter hinge, encoded by two exons, demonstrated higher ADCC capacity compared to other IGHG3 allotypes. This allotype has been observed only in one individual in our dataset. Further, the IGHG3*17 and IGHG3*01m versions of an anti-HIV broadly neutralizing antibody with hinges encoded by three and four exons respectively, did not show consistent differences in Fc-mediated effector functions nor viral neutralization [27]. On the other hand, mutant IGHG3 hinge variants resembling the natural two and three exon allotypes were shown to enhance complement activation and complement-mediated cell lysis as compared to the four exon variant [28, 29]. Thus, functional distinctions between the hinge length variants are plausible, but more work needs to be done with regard to naturally occurring variants.

Multiple disease associations with IgG allotype polymorphisms have been reported in the literature, supporting differential functions of IgG allotypes [14–16]. Given the high level of inter-population diversity at the locus, it is critically important to perform *IGHG* disease association studies in cohorts of sufficiently large sizes and account for population substructure using genome-wide SNP data. The *IGHG* locus is consistently excluded from SNP-based genome-wide association studies (GWAS) due to difficulties in distinguishing the homologous *IGHG* genes, which like the *HLA* class I loci, contain polymorphic sites potentially acquired by gene conversion. Therefore, possible associations with this locus would be missed in these studies. The analysis of the *IGHG* locus should be feasible, however, with the increasing volume of whole exome/genome sequencing data and cautious attention to accurate assembly of sequences. Comparison of data obtained using different methods, as performed in this study, is important for validation and improvement of the genotyping protocols and analyses. This may require parallel analyses of the *IGHG* locus in a larger cohort by both Sanger sequencing and whole exome/genome NGS with proper correction for population stratification, missing in the current study. While Sanger data can improve phasing of short NGS reads, NGS data may help to determine whether primer-annealing regions contain SNPs in Sanger sequencing. These data can be utilized further to develop targeted NGS protocols for *IGHG* typing. In addition, it would be useful to check whether SNP data obtained using genome-wide chips can be applied to impute *IGHG* alleles similar to that for *HLA* genes [30]. This would allow interrogation of the locus in existing and future GWAS. Alternative non-PCR based approaches to analyze long genetic fragments, such as fosmid cloning or nanopore sequencing, may provide the most accurate data, but these methods are difficult to apply in large cohorts.

In summary, we have characterized *IGHG* variation in three distinct populations using gene-specific PCR amplification and Sanger sequencing. Despite its limitations, this work represents a next step towards better understanding of the *IGHG* diversity. Deeper knowledge of the *IGHG* variation and its physiological consequences will provide further insight into understanding antibody function and promote therapy design in the context of population diversity.

## Material and methods

### Human subjects

Blood samples from healthy African American donors (*N* = 94) were obtained previously from a collaboration with the Duke CHAVI 008 A study (AI067854) [31]. Samples from healthy donors of European ancestry (*N* = 95) were derived from the Research Donor Program (RDP) at the Frederick National Laboratory for Cancer Research. South African samples (*N* = 94) were derived from the FRESH cohort, an observational, prospective cohort of high-risk HIV-negative women launched in Durban, South Africa [32]. Specimen collection and sharing were approved by the representative review boards (IRB): Duke University (CHAVI 008 A), Massachusetts General Hospital (FRESH), and National Cancer Institute (RDP). Written informed consent was obtained from all subjects at all study sites and specimen were anonymized by IRB-approved procedures.

### Genotyping

Genomic DNA was extracted using QIAamp DNA Blood Mini Kit (Qiagen) from frozen PBMC pellets, typically obtained from 10 ml of whole blood by Ficoll density gradient centrifugation. Primers were designed for gene-specific PCR amplification and sequencing of *IGHG1*, *IGHG2*, and *IGHG3* exon coding fragments (Tables S8–S9). PCR amplification was performed separately for each primer pair using Platinum Taq polymerase (Thermo Fisher Scientific) according to the manufacturer’s protocol in 10 ul volume reaction with 10–50 ng of genomic DNA and 0.2 uM primers. The cycling parameters were as follows: initial denaturation, 94 °C - 2 min; 35 cycles, 94 °C - 30 sec, T_ann_ (Table S1) - 30 sec, 72 °C - 1 min/kb; final extension, 72 °C - 7 min. Sanger sequencing was performed using standard BigDye protocols and the ABI 3700 instrument (Thermo Fisher Scientific). PCR and sequencing products were purified using AMPure XP and CleanSEQ magnetic beads (Beckman Coulter) according to the manufacturer’s protocol. Sequencing data were analyzed using Sequencher software (Gene Codes). Variability in the number of exons encoding the hinge region was analyzed by PCR fragment size resolution using a LabChip GX instrument (Perkin Elmer). Polymorphic positions within each gene were labeled to denote gene name, exon name and nucleotide position within the exon: for example, G1_CH1_68. The Eu numbering system was used for amino acid positions [33] (www.imgt.org). To avoid variant calling errors due to background noise and sample handling, genotyping was repeated for >10% of samples, including all variants that were present in single individuals. Table S10 contains all genotyping data generated in this study.

### Haplotype analysis

Haplotypes were built using Haploview 4.1 software (www.broad.mit.edu/mpg/haploview/) with the lower threshold of 1%. The software automatically calculates SNP frequency and *p* value for deviation from Hardy-Weinberg equilibrium, which was not significant for any of the SNPs identified in any of the individual populations.

### The 1kGP data and Fst analysis

The *IGHG* exon variant data for the 1kGP-ph3 dataset [21] and the newly assembled high coverage version, 1kGP-30X [22], were extracted from https://www.internationalgenome.org/data-portal/data-collection/grch38 and https://www.internationalgenome.org/data-portal/data-collection/30x-grch38, respectively. The 1kGP genotypes were obtained using DNA from lymphoblastoid cell lines, which are oligoclonal regarding Ig class switching. The data were processed using Plink v1.9.0, BCFtools v1.9 and VCFtools v0.1.16 [34]. The 1kGP-ph3 data were used for haplotype comparison and the F_ST_ analysis. Weir and Cockerham’s F_ST_ [23] values were calculated for nonsynonymous *IGHG* SNPs having a global minor allele frequency >10% using VCFtools v0.1.16. Empirical *p* values were determined on the F_ST_ values of 1000 randomly sampled SNPs across all autosomes from the 1kGP-ph3 data [21] for super-population pairs, including AFR, EUR, and EAS. Bonferroni correction was applied to *p* values to correct for multiple comparisons.

## Supplementary information

Supplemental material

Table S1

Table S10
