# Supplementary material for: Population-specific diversity of the immunoglobulin constant heavy G chain (IGHG) genes
Source: Genes Immun. 2021 Dec 4;22(7-8):327–34. doi: 10.1038/s41435-021-00156-2 (PMC8674132; doi:10.1038/s41435-021-00156-2)
Supplement: Supplementary file 1 — Supplemental material [file 41435_2021_156_MOESM1_ESM.docx]

**A**

1 TCCCAGATCT GAGTAACTCC CAATCTTCTC TCTGCAGAGC TCAAAACCCC ACTTGGTGAC ACAACTCACA CATGCCCACG GTGCCCAGGT AAGCCAGCCC 100

2 ---------C CC-------- ---------- ---------- C--......A -TC-T----- ---C---C-C -G-------- ---------- ----------

3 ---------C CC-------- ---------- ---------- C--......A -TC-T----- ---C---C-C ---------- ---------- ----------

4 ---------C CC-------- ---------- ---------- C--......A -TC-T----- ---C---C-C -G------A- ---------- ----------

1 AGGCCTCGCC CTCCAGCTCA AGGCGGGACA AGAGCCCTAG AGTGGCCTGA GTCCAGGGAC AGGCCCCAGC AGGGTGCTGA CGCATCCACC TCCA 194

2 ---------- ---------- ----A----- ---------- ---------- ---------- ---------- ---------- ---G------ ----

3 ---------- ---------- ---------- ---------- ---------- ---------- ---------- ---------- ---------- ----

4 ---------- ---------- ----A----- G-T------- ---------C A--------- ---T-----T C--------- -A----TG-- ----

**B**

**
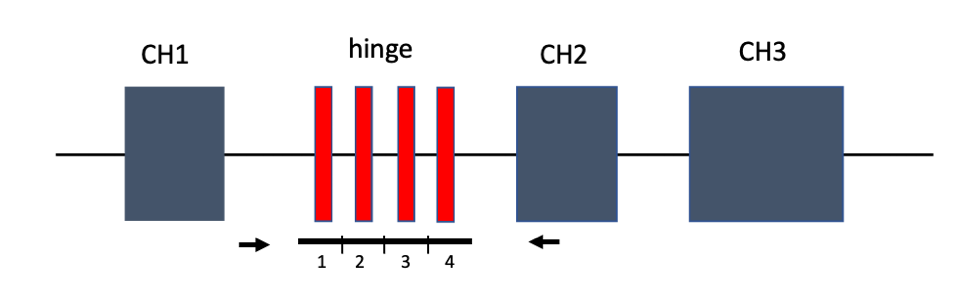
**

Figure S1. (A) Alignment of the four consecutive repetitive elements in the *IGHG3* genomic fragment encompassing the hinge exons (ENSG00000211897, [www.ensembl.org](http://www.ensembl.org)). Exon sequences within the repetitive elements are shown in red. (B) Schematic representation of the *IGHG3* exon-intron struture and location of primers used for PCR to determine the hinge exon copy number. The repetitive region, consisting of the four elements, is marked by black line; the primers, which anneal outside of the repetitive region, are shown as black arrows.

**161 168 311 313**

IGHG1*01 **g**cctccc**g**tgctggactccgacggctccttcttcctctacagcaagctcaccgtggacaagagcaggtggcagcaggggaacgtcttctcatgctccgtgatgcatgaggctctgcacaaccactacacgcagaagagcctctccctgtc**t**c**c**gggtaaa

IGHG1*02 ----------------------------------------------------------------------------------------------------------------------------------------------------------------

IGHG1*03 ---------------------------------------t--------------------------------------------------------------------------------------------------------------c---------

IGHG1*04 ----------------------------------------------------------------------------------a-----------------------------------------------------------------------------

IGHG1*05 ---------------------------------------------------------------------------------------------------------------------------------a------------------------------

IGHG1*06 ---------------------------------------t--------------------------------------------------------------------------c-----------------------------------c---------

IGHG1*07 --------------------------------------------------------------------------------------------------------------g-------------------------------------------------

IGHG1*08 ----------------------------------------------------------------------------------------------------------------------------------------------------------------

IGHG1*09 ----------------------------------------------------------------------------------------------------------------------------------------------------------------

IGHG1*10 ----------------------------------------------------------------------------------------------------------------------------------------------------------------

IGHG1*11 ----------------------------------------------------------------------------------------------------------------------------------------------------------------

IGHG1*12 ----------------------------------------------------------------------------------------------------------------------------------------------------------------

IGHG1*13 ----------------------------------------------------------------------------------------------------------------------------------------------------------------

IGHG1*14 ----------------------------------------------------------------------------------------------------------------------------------------------------------------

IGHG2*01 a------a--------------------------------------------------------------------------------------------------------------------------------------------------------

IGHG2*02 a------a-------------------------------------------------------------------------------------------------------------------------a------------------------------

IGHG2*03 a------a--------------------------------------------------------------------------------------------------------------------------------------------------------

IGHG2*04 a------a--------------------------------------------------------------------------------------------------------------------------------------------------------

IGHG2*05 -------a--------------------------------------------------------------------------------------------------------------------------------------------------------

IGHG2*06 a------a-------------------------------------------------------------------------------------------------------------------------a------------------------------

IGHG2*07 a------a--------------------------------------------------------------------------------------------------------------------------------------------------------

IGHG2*08 -------a--------------------------------------------------------------------------------------------------------------------------------------------------------

IGHG2*09 a------a--------------------------------------------------------------------------------------------------------------------------------------------------------

IGHG2*10 a------a--------------------------------------------------------------------------------------------------------------------------------------------------------

IGHG2*11 -------a--------------------------------------------------------------------------------------------------------------------------------------------------------

IGHG2*12 a------a--------------------------------------------------------------------------------------------------------------------------------------------------------

IGHG2*13 -------a--------------------------------------------------------------------------------------------------------------------------------------------------------

IGHG2*14 a------a--------------------------------------------------------------------------------------------------------------------------------------------------------

IGHG2*15 -------a--------------------------------------------------------------------------------------------------------------------------------------------------------

IGHG2*16 -------a-------------------------------------------------------------------------------------------------------------------------a------------------------------

IGHG2*17 -------a-------------------------------------------------------------------------------------------------------------------------a------------------------------

IGHG3*01 -------a--------------------------------------------------------------------------a---------------------------------------g--t----------------------------------

IGHG3*02 -------a-------*************************************************************************************************************************************************

IGHG3*03 --------------------------------------------g---a------------------------g-------t----------------------------------------g--t----------------------------------

IGHG3*04 -------a--------------------------------------------------------------------------a---------------------------------------g--t----------------------------------

IGHG3*05 -------a--------------------------------------------------------------------------a---------------------------------------g--t----------------------------------

IGHG3*06 -------a--------------------------------------------------------------------------a---------------------------------------g--t----------------------------------

IGHG3*07 -------a--------------------------------------------------------------------------a---------------------------------------g--t----------------------------------

IGHG3*08 -------a--------------------------------------------------------------------------a---------------------------------------g--t----------------------------------

IGHG3*09 -------a-------------------------------------------t------------------------------a---------------------------------------g--t----------------------------------

IGHG3*10 -------a--------------------------------------------------------------------------a---------------------------------------g--t----------------------------------

IGHG3*11 -------a--------------------------------------------------------------------------a---------------------------------------g--t----------------------------------

IGHG3*12 -------a--------------------------------------------------------------------------a---------------------------------------g--t----------------------------------

IGHG3*13 a------a-----------------------------------------------------------------g--------a---------------------------------------g--t----------------------------------

IGHG3*14 -------a--------------------------------------------------------------------------a---------------------------------------g-------------------------------------

IGHG3*15 -------a--------------------------------------------------------------------------a---------------------------------------g-------------------------------------

IGHG3*16 -------a--------------------------------------------------------------------------a---------------------------------------g-------------------------------------

IGHG3*17 ----------------------------------------------------------------------------------a-----------------------------------------------------------------------------

IGHG3*18 ----------------------------------------------------------------------------------a-----------------------------------------------------------------------------

IGHG3*19 ----------------------------------------------------------------------------------a-----------------------------------------------------------------------------

IGHG3*20 -------a--------------------------------------------------------------------------a---------------------------------------g-------------------------------------

IGHG3*21 -------a--------------------------------------------------------------------------a---------------------------------------g--t----------------------------------

IGHG3*22 -------a--------------------------------------------------------------------------a-----------------------------------------------------------------------------

IGHG3*23 ----------------------------------------------------------------------------------a-----------------------------------------------------------------------------

IGHG3*24 -------a--------------------------------------------------------------------------a---------------------------------------g-------------------------------------

IGHG3*25 -------a--------------------------------------------------------------------------a---------------------------------------g-------------------------------------

IGHG3*26 -------a--------------------------------------------------------------------------a---------------------------------------g--t----------------------------------

IGHG3*27 -------a--------------------------------------------------------------------------a---------------------------------------g-------------------------------------

IGHG3*28 -------a--------------------------------------------------------------------------a---------------------------------------g--t----------------------------------

IGHG3*29 -------a--------------------------------------------------------------------------a---------------------------------------g-------------------------------------

IGHG4*01 --------------------------------------------g---a------------------------g-------t-----------------------------------------------a----------------------t-------

IGHG4*02 --------------------------------------------g---a------------------------g-------t----------------------------------------------------------------------t-------

IGHG4*03 -------------------------------------------------------------------------g------------------------------------------------------------------------------t-------

IGHG4*04 --------------------------------------------g----------------------------g-------t-----------------------------------------------a----------------------t-------

IGHG4*05 a-------------------------------------------g----------------------------g-------t-----------------------------------------------a----------------------t-------

IGHG4*06 --------------------------------------------g----------------------------g-------t-----------------------------------------------a----------------------t-------

IGHG4*07 --------------------------------------------g---a------------------------g-------t-----------------------------------------------a----------------------t-------

IGHG4*08 --------------------------------------------g---a------------------------g-------t-----------------------------------------------a----------------------t-------

Fig. S2. Nucleotide alignment of CH3 exon fragments (nt. 161-320) for IMGT *IGHG* alleles. Positions of four SNPs differentially detected in the 1kGP datasets and the current study are shown in red. 1kGP-ph3 does not detect polymorphism at position 161 in *IGHG2* and position 311 in *IGHG1*. 1kGP-30X shows variation in *IGHG1 CH3* at position 168 (G>A, gmaf 24%) and *IGHG2* shows polymorphism at position 313 (C>T, gmaf 19%). Position numbering is based on position within the CH3 exon.


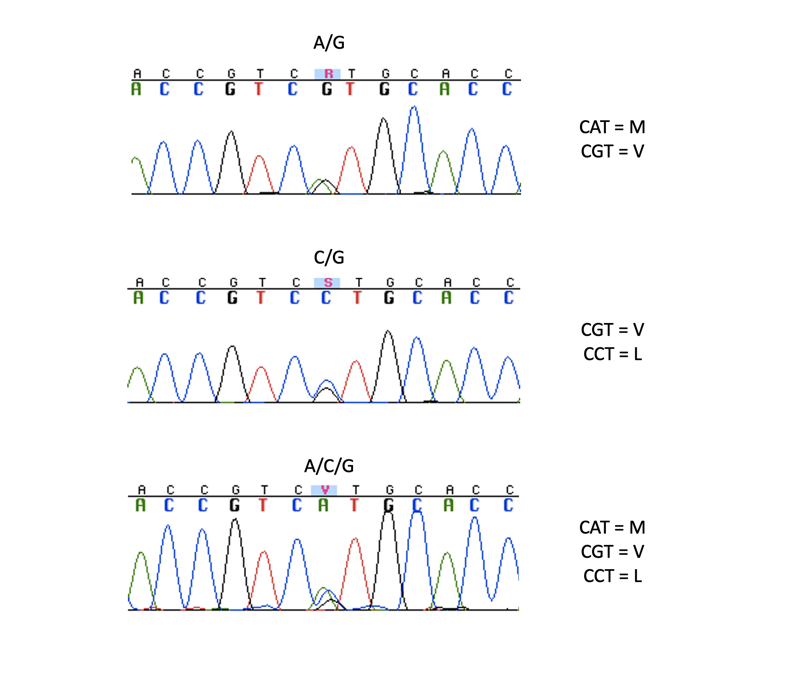


Fig. S3. Trimorphism in the *IGHG2* gene. Sequence electrophoregram depicting heterozygosity for rs113678609 (aa pos. 309, nt position 231 in CH2 exon) in three different individuals. The bottom electrophoregram shows the presence of three nucleotide variants at the SNP position. Corresponding amino acid codons are shown on the right. The PCR product was confirmed to be gene-specific based on the characteristic *IGHG2* sequence of the CH2 exon, which includes a 3 bp deletion that is not present in *IGHG1*, *IGHG3*, or *IGHG4*.


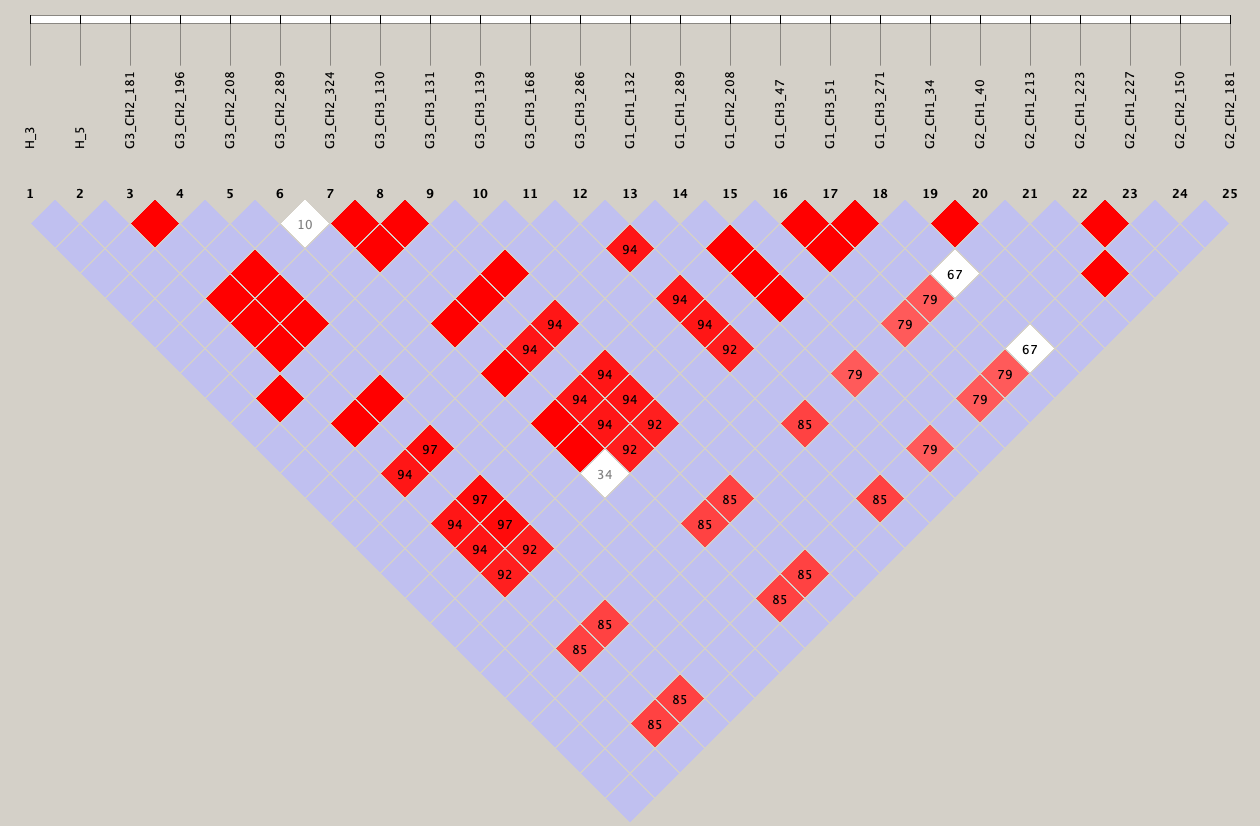


EA


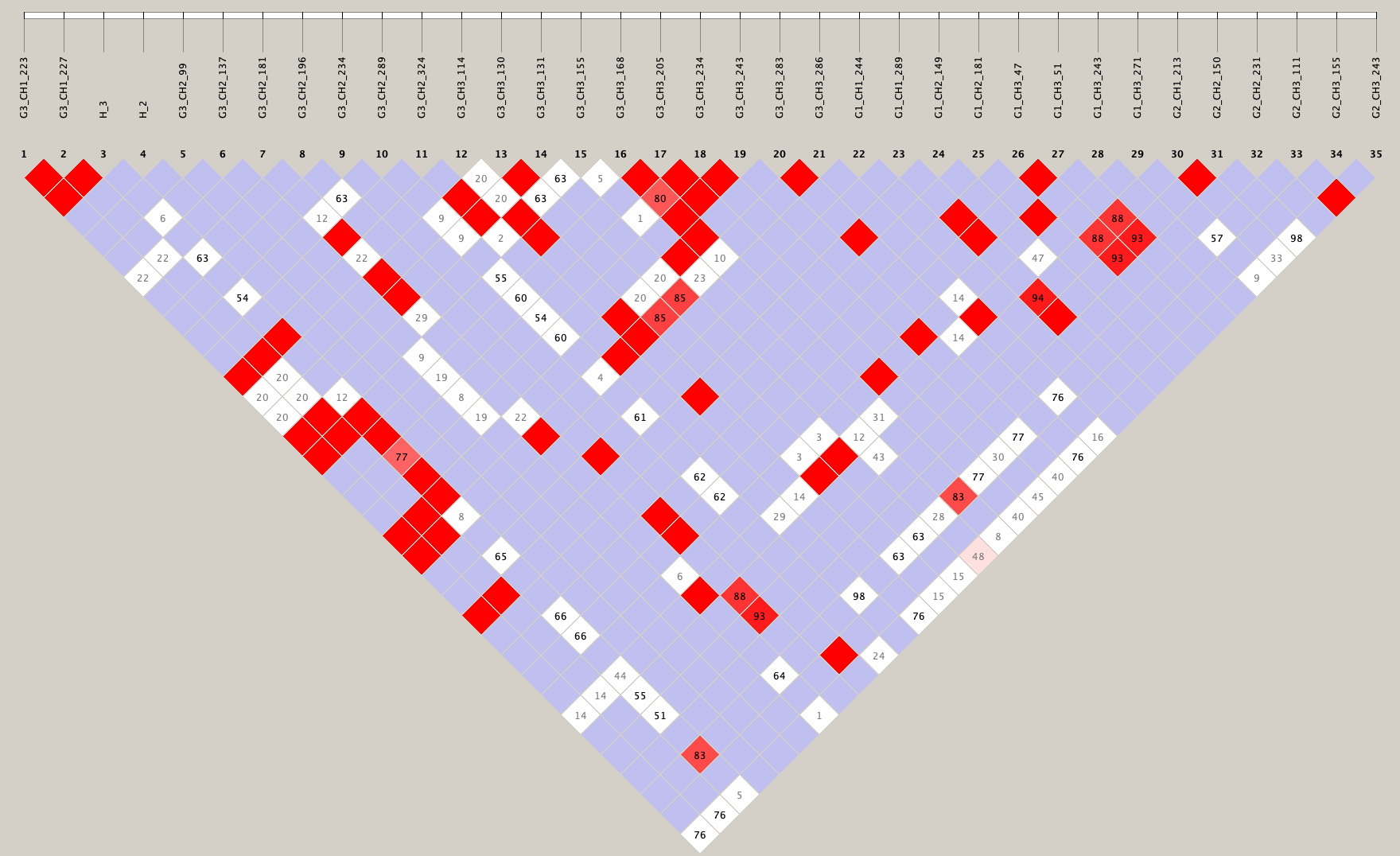


AA


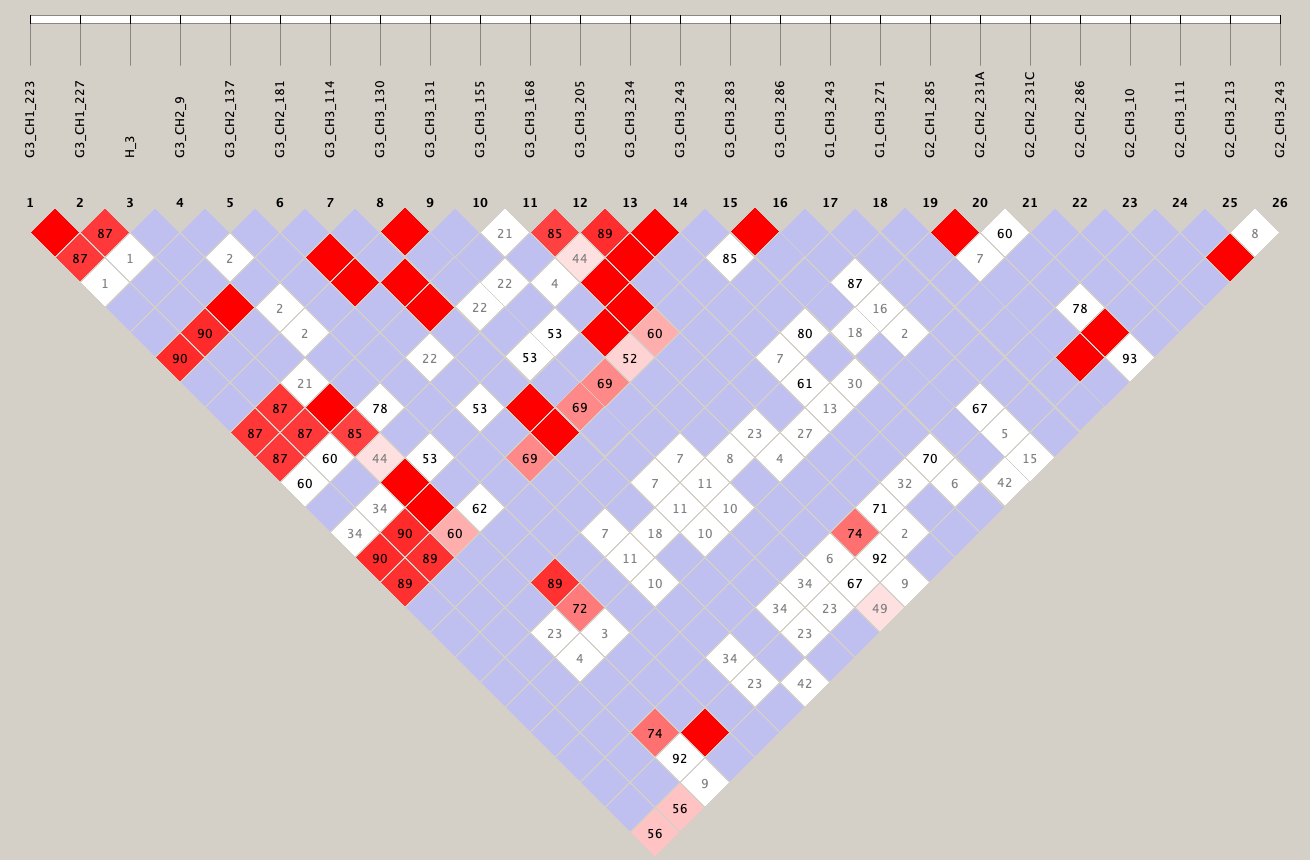


SA

Fig. S4. LD plots generated by Haploview for amino acid changing variants encompassing *IGHG3*, *IGHG1*, and *IGHG2* in the three populations tested. Colors reflect two statistical estimates of LD: D’ and LOD (logarithm of the odds) score: bright red - D’ = 1, LOD 2; shades of pink/red - D’ < 1, LOD 2; white - D’ < 1, LOD < 2; blue – D’ = 1, LOD < 2. Numbers correspond D’ values multiplied by 100, empty red boxes correspond to D’ = 1. H_2, H_3, H_5 – *IGHG3* hinge exon number variants (two, three, and five exons variants). SNPs positions within the corresponding genes and exons are shown on top (e.g. G3_CH1_223 is nt position 223 in the *IGHG3* CH1 exon). SNP information can be found in Table S1.


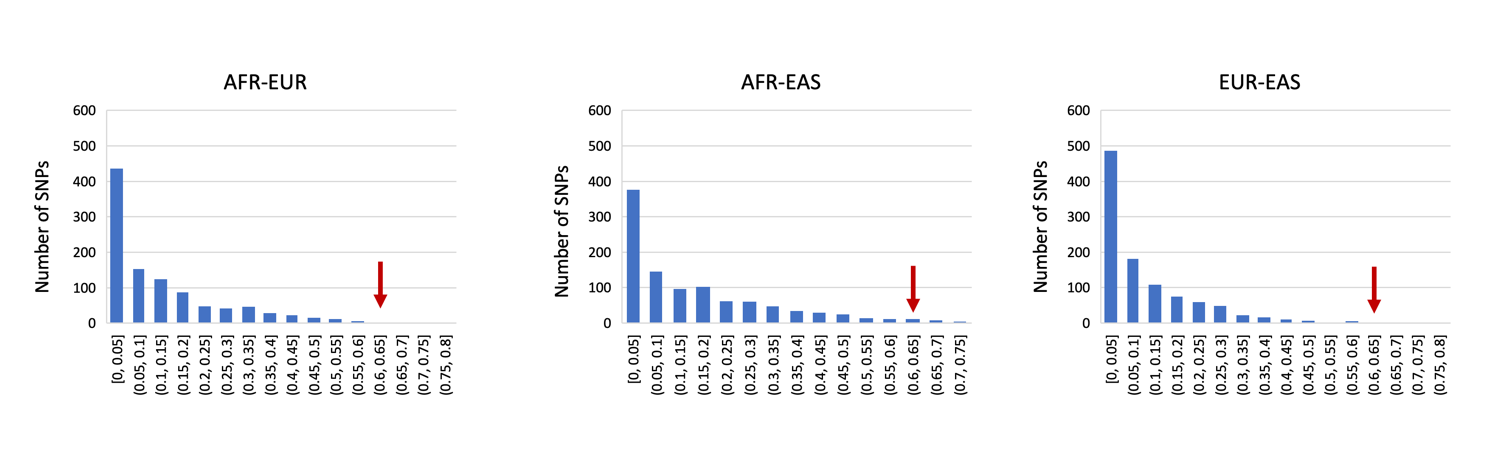


Fig. S5. Pairwise F_ST_ distribution of a sample of 1000 SNPs across autosomes between pairs of the three super-populations from 1kGP-ph3. All sampled SNPs are bi-allelic and of global minor allele frequency 10%. The red arrows indicate the positions of the significant estimated F_ST_ for the SNPs shown in Table 5.

Table S1. IGHG SNPs and frequencies identified in the current study and 1kGP datasets (Excel file with the three worksheets – Current study, 1kGP datasets, Abbreviations and notes).

Table S2. IGHG1 haplotypes^1^.

^1^Haplotypes with frequency >1% were estimated using Haploview. Numerical allele names correspond to the IMGT nomenclature, and putative novel alleles are labeled with letter “n”. Non-synonymous SNPs are shown in red. SNP positions within the corresponding exons are shown in the header row. Chromosomal positions, and SNP frequencies can be found in Table S1. Allotypes are given the name of the most frequent allele encoding the identical protein variant.

Table S3. IGHG2 haplotypes^1^.

^1^ See footnote to Table S2.

Table S4. IGHG3 haplotypes^1^.

^1^ See footnote to Table S2.

Table S5. Additional IGHG3 allotypes estimated in the 1kGP-ph3 super-populations^1^.

^1^ The allotypes IGHG3*1K1-*1K17 were present only in the 1kGP dataset. IGHG3*01 is shown for reference. Amino acid positions are in accordance with Eu numbering. Hinge exon number (H) and polymorphism at amino acid position 419 are missing from 1kGP-ph3 dataset.

Table S6. IGHG3_IGHG1_IGHG2 haplotype frequencies estimated in the current study^1^.

^1^ The allotypes represent unique amino acid sequences designated as shown in Tables 1-3. Gm haplotypes were defined according to Lefranc and Lefranc ^7^.

Table S7. Gm haplotype frequencies estimated in the current study^1^.

^1^ Gm haplotypes were defined according to Lefranc and Lefranc ^7^.

Table S8. PCR amplification primers.

Table S9. Sequencing primers.

^1^Does not always work in individuals of African descent due to a downstream indel polymorphism.
^2^May miss some alleles in Asian populations.

Table S10 - Genotyping data generated in the current study (Excel file).
